# Protein-Aided Synthesis of Copper-Integrated Polyaniline Nanocomposite Encapsulated with Reduced Graphene Oxide for Highly Sensitive Electrochemical Detection of Dimetridazole in Real Samples

**DOI:** 10.3390/polym16010162

**Published:** 2024-01-04

**Authors:** Kartik Behera, Bhuvanenthiran Mutharani, Yen-Hsiang Chang, Monika Kumari, Fang-Chyou Chiu

**Affiliations:** 1 Department of Chemical and Materials Engineering, Chang Gung University, Taoyuan 333, Taiwan; b.kartik1991@gmail.com (K.B.); mutharani@gmail.com (B.M.); 2 Department of General Dentistry, Chang Gung Memorial Hospital, Taoyuan 333, Taiwan; chy4d25@cgmh.org.tw; 3 Institute of Cellular and Organismic Biology, Academia Sinica, Taipei 115, Taiwan; mkumari.biotech@gmail.com

**Keywords:** biomimetic mineralization, bovine serum albumin, nanocomposite, electrocatalyst, antibiotic dimetridazole detection, biological samples

## Abstract

Dimetridazole (DMZ) is a derivative of nitroimidazole and is a veterinary drug used as an antibiotic to treat bacterial or protozoal infections in poultry. The residues of DMZ cause harmful side effects in human beings. Thus, we have constructed a superior electrocatalyst for DMZ detection. A copper (Cu)-integrated poly(aniline) (PANI) electrocatalyst (PANI-Cu@BSA) was prepared by using a one-step method of biomimetic mineralization and polymerization using bovine serum albumin (BSA) as a stabilizer. Then, the synthesized PANI-Cu@BSA was encapsulated with reduced graphene oxide (rGO) using an ultrasonication method. The PANI-Cu@BSA/rGO nanocomposite had superior water dispersibility, high electrical conductivity, and nanoscale particles. Moreover, a PANI-Cu@BSA/rGO nanocomposite-modified, screen-printed carbon electrode was used for the sensitive electrochemical detection of DMZ. In phosphate buffer solution, the PANI-Cu@BSA/rGO/SPCE displayed a current intensity greater than PANI-Cu@BSA/SPCE, rGO/SPCE, and bare SPCE. This is because PANI-Cu@BSA combined with rGO increases fast electron transfer between the electrode and analyte, and this synergy results in analyte–electrode junctions with extraordinary conductivity and active surface areas. PANI-Cu@BSA/rGO/SPCE had a low detection limit, a high sensitivity, and a linear range of 1.78 nM, 5.96 μA μM^−1^ cm^−2^, and 0.79 to 2057 μM, respectively. The selective examination of DMZ was achieved with interfering molecules, and the PANI-Cu@BSA/rGO/SPCE showed excellent selectivity, stability, repeatability, and practicability.

## 1. Introduction

Dimetridazole (DMZ) is a derivative of nitroimidazole, a veterinary drug used as an antibiotic to treat bacterial or protozoal infections in poultry [1]. Residues of DMZ cause harmful side effects in human beings, such as carcinogenicity, genotoxicity, and mutagenicity, owing to its strong carcinogenic teratogenicity ring structure [2,3,4]. Specifically, it has a carcinogenic effect on the human respiratory system. A higher dose of DMZ might cause lung damage, respiratory problems, and cancer in patients, and it could be associated with kidney damage, emphysema, and chronic bronchitis [3]. Regarding safety disquiets, DMZ has been prohibited in animal feed production in several countries, including the European Union, the USA, Canada, and China [5,6]. Consequently, techniques for detecting DMZ are of prodigious interest in scientific and medical research. Various analytical techniques such as gas chromatography, polarography, immunoassay, liquid chromatography–mass spectrometry, capillary electrophoresis, and electrochemical (EC) techniques can be utilized for this purpose [7,8]. Among these, EC methods are the most extensively applied, as they often provide rapid, cost-effective detection and are more sensitive and effective than other methods [9,10,11]. Therefore, there is an urgent need to develop a highly selective and sensitive electrode modifier for efficient DMZ detection. 

Nanostructured materials (NSMs) have drawn great interest and thus are called “a wonder” in the field of electrochemistry [12,13]. A strategy that has been developed involves utilizing NSMs to construct electrochemical sensors (ECSs), upgrading electrode catalytic attributes, escalating the sensor surface area, and achieving nanoscale sensors [14,15]. In recent years, various metal nanoparticle (MNP)-modified ECSs have been reported, such as Ag NPs, Au NPs, Pd NPs, and Pt NPs [16]. In contrast with noble NPs, copper nanoparticles (Cu NPs) have gained attention for their versatile applications due to their environmental friendliness, cost-effectiveness, and strong electrical and catalytic attributes [17,18]. Recently, composites of MNPs and conductive polymers (CPs) have gained growing consideration not only due to scientific attraction but also owing to their practicable implementations in electrocatalysts and sensors/biosensors [19,20]. Nanocomposite formation is important for electrocatalysis and is a key novelty of this manuscript [21]. Sensor studies have shown that immobilization of MNPs on conductive polymers (e.g., polyaniline (PANI), poly(3,4-ethylene dioxythiophene) (PEDOT), polypyrrole (PPy), etc.) further enhances EC activity [22]. In this case, PANI-based MNP catalysts are typically considered very useful materials for EC utilization [23].

PANI is one of the CPs that is extensively applied as an electrode material for sensor applications due to its excellent conductivity, flexibility, easy control during preparation, high stability, high theoretical specific capacitance, and ease in creating composites with other materials [24,25]. Accordingly, the MNP-immobilized PANI-modified electrode was employed in various electrochemical sensor applications [26,27]. Hence, PANI/Cu NPs might be the appropriate choice for sensor applications. Yet, the majority of the stated PANI-based MNP electrocatalysts unveil acute accumulation and a huge particle size and lack dispersal in aqueous solutions, which significantly confines their catalytic applications. Consequently, the creation of stable, functional, and small PANI-Cu NPs with excellent dispersibility for sensor applications is intriguing.

Biogenic-mineralization machinery is commonly used (peptide, protein, polymer, collagen, etc.) as the template to successfully regulate the control of organisms by controlling bio-based molecules [28,29]. Bovine serum albumin (BSA) protein-assisted biomineralized MNPs have enticed considerable heed due to their diverse advantages comprising biocompatibility, eco-friendly processing, excellent reproducibility, and efficient stability [30]. Several studies reported that the BSA can be utilized to avert MNPs aggregations [31]. Recent research has also utilized commercially available BSA protein to synthesize various ultra-small MNPs for biomedical applications [32,33]. Therefore, we hypothesize that the high stability and aquatic solubility of BSA protein and the existence of different binding sites in its molecular structure would be vital factors in stabilizing PANI-Cu NPs and preventing their accumulation to improve their EC sensing performance. Intriguingly, BSA also improves material adhesion to the electrode surface, preventing material detachment from the electrode surface. BSA-Cu NPs, stabilized by BSA for long-term stability, not only improve biosensors with higher sensitivity but also serve as a biocompatible matrix facilitating effective biomolecule immobilization. The inherent features of Cu NPs contribute to signal amplification, improving detection limits [34]. Therefore, in this work, we adopted the biomineralization strategy for the synthesis of PANI-Cu@BSA. 

In addition, various research groups have described that the combination of CP-based MNPs with graphene (GR) prominently upgrades the electrical conductivity, material stability, and mechanical strength of the final nanocomposites [35,36]. GR and its derivatives are the focus of interest for ECS applications because of their distinctive attributes. Reduced GO (rGO) is particularly favored for various EC applications due to its simplistic and scalable manufacture, low cost, large surface area, and superior electrical conductivity [37]. rGO exhibits oxygen-containing groups and a large number of lattice defects, which can effectively provide an active site to facilitate the deposition of polymers/polymer-MNPs [38]. Therefore, embedding a PANI-Cu nanostructure on the rGO surface can greatly improve the electrical properties and promote EC sensitivity. These aforementioned factors motivated us to synthesize the PANI-Cu@BSA/rGO ternary nanocomposite to achieve better EC performance.

In the present work, a nascent PANI-Cu@BSA/rGO ternary nanocomposite was devised as part of the advancement of an enhanced DMZ EC sensor. The ECS was found to offer excellent DMZ detection with great specificity, sensitivity, a low-detection limit, good linearity, and reproducibility. The synergistic effect amid the PANI-Cu@BSA and rGO led to an improved electron migration of the electrode-sensitive interfaces. In addition, the newly constructed PANI-Cu@BSA/rGO was successfully employed in the selective detection of DMZ in real human, rat blood serum, and egg samples with satisfactory outcomes. 

## 2. Materials and Methods

### 2.1. Materials

Bovine serum albumin (BSA) protein, copper chloride (CuCl_2_), sodium hydroxide (NaOH), hydrochloric acid (HCl), ammonium persulfate (APS), aniline (ANI), dimetridazole (DMZ), azathioprine (AZP), carbendazim (CRD), dexamethasone (DEX), irinotecan (IRT), lopinavir (LPN), and nitrofurazone (NFZ) were obtained from Sigma Aldrich (Taipei, Taiwan). Throughout this work, all materials used were of analytical quality and were used as received. 

### 2.2. Preparation of PANI-Cu@BSA and PANI-Cu@BSA/rGO Nanocomposite

**Scheme 1:**
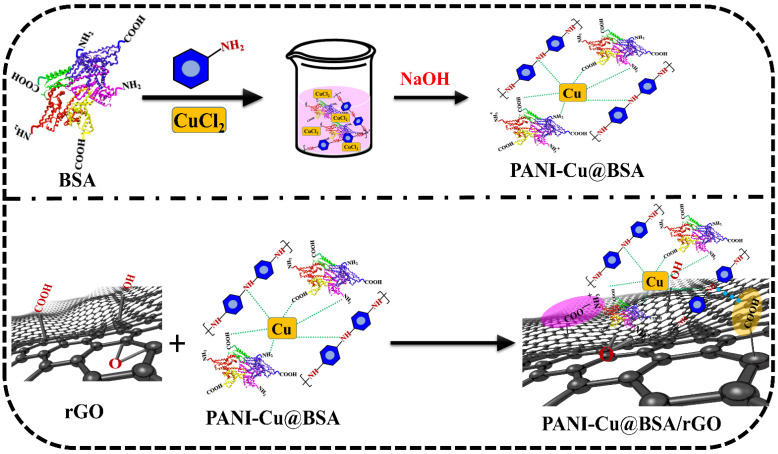
Pictorial representation of the synthesis process of the PANI-Cu@BSA/rGO.

PANI-Cu@BSA was prepared by using a one-pot method. Initially, 120 mg of BSA was dissolved in 18 mL of double distilled (DD) water using a magnetic stirrer for 20 min. Subsequently, HCl (30 µL) and CuCl_2_ solutions (25 mM, 1 mL) were added to the BSA solution, resulting in a transparent solution. Then, 120 µL of ANI was added to this reaction mixture and thoroughly mixed in an ice bath (5 °C) using a magnetic stirrer for 30 min. Polymerization of ANI was started after adding APS (45 mg). After 15 min, the solution changed to a black color because of the ANI polymerization and was kept aside for 24 h. Then, a NaOH solution (2 M) was added to the black solution to adjust the pH value of 12. A modified Hummer’s method [36,39] was used to synthesize reduced graphene oxide (rGO) from graphene oxide (GO). To prepare the PANI-Cu@BSA/rGO nanocomposite, 5 mg of the as-synthesized rGO was dispersed in 50 mL of DD water and ultrasonicated for 15 min. Then, 0.3 g of PANI-Cu@BSA powder was added to the rGO suspension under continuous ultrasonication. After continuing the reaction for 1 h, the PANI-Cu@BSA/rGO (final product) precipitate was obtained by using centrifugation and was rinsed many times with DD water. Scheme 1 displays the schematic illustration of the synthesis process of the PANI-Cu@BSA/rGO.

### 2.3. Characterization

An X-ray photoelectron spectroscopy investigation was conducted using a Theta Probe spectrometer to determine the chemical oxidation state and composition of the rGO and PANI-Cu@BSA/rGO/SPCE samples. The morphology of the fabricated samples was examined using both a transmission electron microscope (TEM) (Jeol JEM-2000EX II, Tokyo, Japan) and a field emission scanning electron microscope (FESEM) (Jeol JSM-7500F, Hitachi High-Technologies Corp., Tokyo, Japan). The energy-dispersive X-ray (EDX) results were obtained by using FESEM with an accelerating voltage of 5 kV. The UV–vis absorption spectra of the prepared samples were obtained using a UV–vis spectrophotometer (JASCO, V-650, Tokyo, Japan) with a wavelength range of 200 to 900 nm. A Bruker TENSOR 27 IR spectrometer was used to obtain the Fourier-transform infrared (FTIR) spectra within a wavenumber range of 400–4500 cm^−1^. The particle size and zeta potential of the samples were evaluated with a ZetaSizer^®^ Nano ZS 90 (Malvern Instruments, UK) working with a 633 nm laser at a backscattering angle of 173° and at a temperature of 25 °C. Before taking measurements, the samples were diluted 50-fold with distilled water and allowed to equilibrate at 25 °C for 300 s to prevent any convection-induced artifacts. Raman spectra were obtained by using an Andor SOLIS instrument (Andor, UK) with a 633 nm laser.

### 2.4. Electrode Fabrication and Electrochemical Experiments

To achieve improved dispersion, 4 mg of PANI-Cu@BSA/rGO was dissolved in 1 mL of DD water and sonicated for 5 h prior to fabricating the electrodes. Subsequently, 6 µL of the uniformly dispersed PANI-Cu@BSA/rGO solution was applied onto the screen-printed carbon electrode (SPCE) surface, coded as PANI-Cu@BSA/rGO/SPCE. A similar procedure was also used for the other modified SPCE samples such as rGO/SPCE and PANI-Cu@BSA/SPCE. Electrochemical measurements were performed by using a CHI650A analyzer together with a three-electrode system. The rGO-, PANI-Cu@BSA-, and PANI-Cu@BSA/rGO-modified SPCEs were used as the working electrode, platinum (Pt) wire was used as the auxiliary electrode, Ag/AgCl was used as the reference electrode, and 0.1 M phosphate buffer saline (PBS) was used as a supporting electrolyte. All potentials were reported against the Ag/AgCl. 

## 3. Results and Discussion

### 3.1. Morphological and Structural Characteristics of PANI-Cu@BSA/rGO Nanocomposite

PANI-Cu@BSA was prepared using the “one-pot” method at a mild temperature. An aqueous dispersion polymerization reaction was carried out to acquire PANI, and BSA was used as a reaction scaffold and stabilizer [28]. PANI polymerization was initiated using the oxidizing agent APS. As a result, the reaction mixture’s color changed to black, which confirmed the development of PANI. The Cu molecules were efficiently connected to BSA and PANI cavities because of their exceptional compatibility during the polymerization reaction. The pH of the solution was modified to alkaline (12) for biomimetic mineralization. The structural change in BSA from its 3D folded form to an unfolded form occurred when the pH was raised to 12 (alkaline) [40,41]. Furthermore, the hydrolysis of CuCl_2_ into CuO/Cu(OH)_2_ occurred under alkaline conditions; consequently, Cu molecules were developed in enormous BSA and PANI cavities. Then, the rGO was encapsulated with PANI-Cu@BSA by using an ultrasonication process. Lastly, PANI-Cu@BSA/rGO was developed by filtering the liquid. Improved dispersion stability and EC properties are advantageous. 

**Figure 1:**
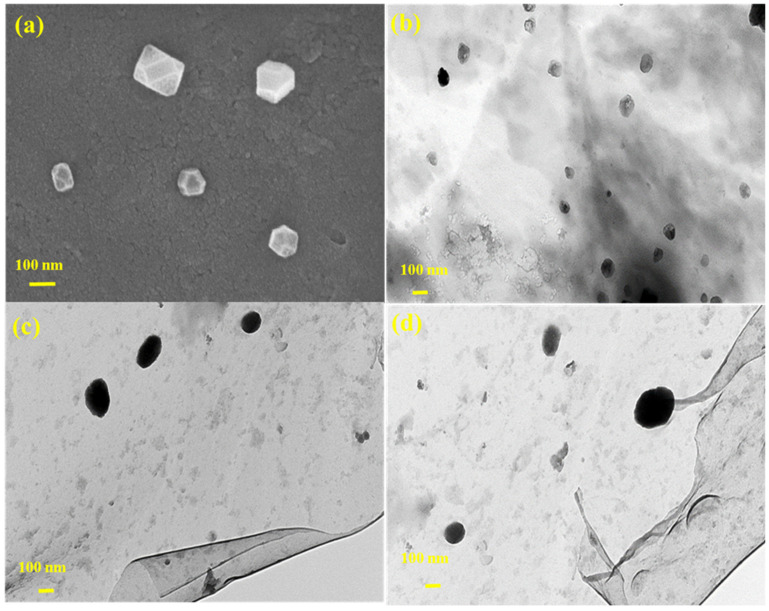
(**a**) FE-SEM image of PANI-Cu@BSA and TEM images of (**b**) PANI-Cu@BSA and (**c**,**d**) PANI-Cu@BSA/rGO.

**Figure 2:**
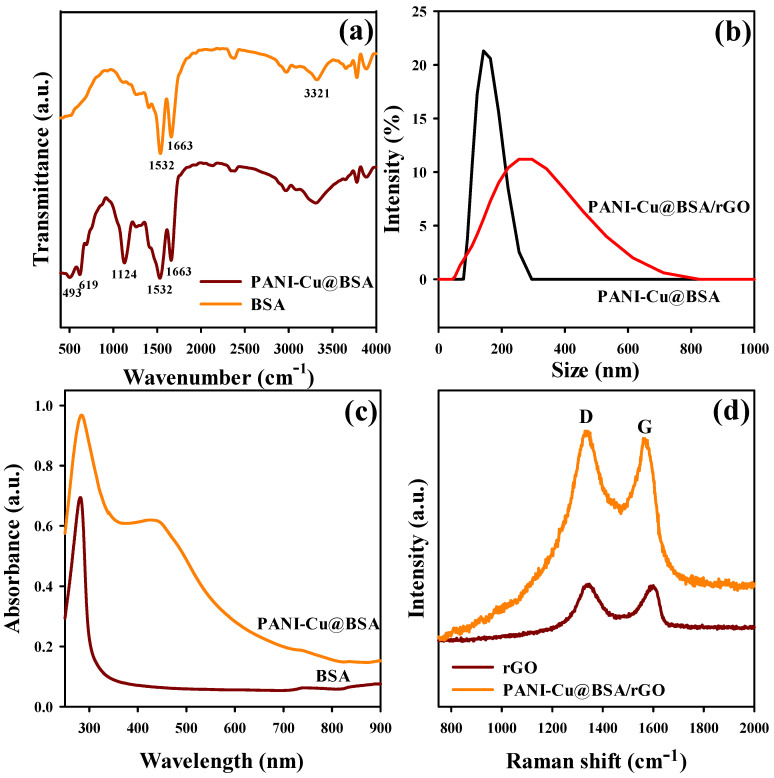
(**a**) FTIR spectra of BSA and PANI-Cu@BSA, (**b**) zeta of PANI-Cu@BSA and PANI-Cu@BSA/rGO, (**c**) UV spectra of BSA and PANI-Cu@BSA, and (**d**) Raman spectra of rGO and PANI-Cu@BSA/rGO.

FESEM and TEM images (Figure 1a–d) show that the fabricated PANI-Cu@BSA/rGO had an irregular/cubical-like morphology with an average diameter of 100 nm. However, DLS measurements revealed that the size of PANI-Cu@BSA/rGO (220 nm) was larger than that of PANI-Cu@BSA (145 nm). However, the average size obtained from zeta measurements was larger than that of TEM images because of the water solubility of BSA. The larger average size obtained from zeta measurements compared to TEM images was due to a combination of the hydrated layer formed by water-soluble BSA and the improved dispersion of PANI-Cu@BSA/rGO particles in the solution. As a result of the substantial negative charge on PANI-Cu@BSA/rGO, the zeta potential of the compound was measured to be 32 mV, enabling excellent dispersion of PANI-Cu@BSA/rGO in water (Figure 2b). Figure S1 shows the TEM image of nanosheets of rGO. The EDX electron images and the mapping images of PANI-Cu@BSA/rGO are shown in Figure S2a–f. 

The FTIR measurements in Figure 2a quantify the nature of molecular configuration and chemical bonding of the samples. BSA showed characteristic transmittance peaks at 1392 cm^−1^ for C–N stretching vibration; 1532 cm^−1^ for amide II (N–H bending vibration), and 1663 cm^−1^ for amide I (C=O stretching vibration) [28]. PANI-Cu@BSA and BSA both exhibited these peaks in their FTIR spectra. Moreover, PANI-Cu@BSA showed an additional strong peak at 1124 cm^−1^ due to the aromatic C–H in-plane bending vibration of PANI. Furthermore, the peak at 619 cm^−1^ was due to the Cu–O vibration in PANI-Cu@BSA [42]. Similar findings were previously reported [28,42]. 

BSA showed maximum UV absorbance at 281 nm. In the composite, Cu could be detected by its absorbance at 416 nm (Figure 2c). Figure 2d presents the Raman results of rGO and PANI-Cu@BSA/rGO composite. The spectra exhibited distinct peaks at 1336 cm^−1^ and 1596 cm^−1^ attributed to the D and G bands of rGO, respectively. The D band represents the numerous defects present at the periphery of rGO and amorphous structures, while the G band signifies the E_2g_ mode of phonon vibrations that occur within carbon materials bonded via sp^2^ [39]. The PANI-Cu@BSA/rGO material exhibited a shift of 26 cm^–1^ in the G band compared to rGO. This was due to the charge transfer between rGO and Cu as well as the π–π interaction between PANI and rGO [39]. The D band was not shifted in the Raman spectra of PANI-Cu@BSA/rGO.

**Figure 3:**
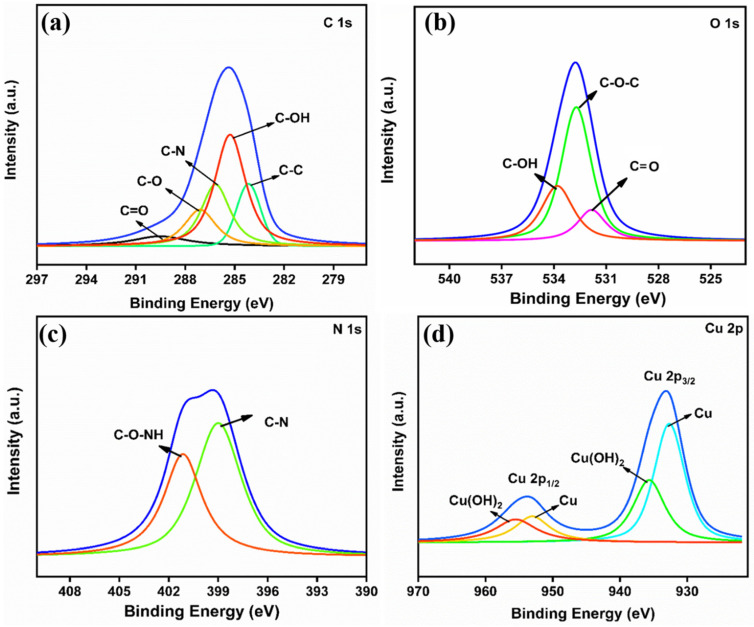
Core-level XPS spectrum of (**a**) C 1s, (**b**) O 1s, (**c**) N 1s, and (**d**) Cu 2p for PANI-Cu@BSA/rGO.

Furthermore, the elemental composition of the PANI-Cu@BSA/rGO nanocomposite was evaluated by utilizing the XPS analysis. The high-resolution XPS spectra of C 1s (Figure 3a) exhibited the binding energies at 284.2, 285.0, 285.6, 287.1, and 288.9 eV, which correlated with the C-C, C-OH, C-N, C-O, and C=O groups in PANI-Cu@BSA/rGO, respectively. The core-level O 1s spectrum (Figure 3b) could be deconvoluted into three peaks at 531.6, 532.2, and 533.5 eV, which was ascribed to C=O, C-O-C, and C-OH, respectively. Figure 3c displays the XPS spectra of N 1s, showing two binding energies at 400.2 and 401.3 eV for C-N and C-O-NH, respectively. In the high-resolution Cu 2p spectrum (Figure 3d), two characteristic Cu peaks were observed at the binding energies of 932.5 and 952.4 eV, which were ascribed to Cu 2p_3/2_ and Cu 2p_1/2_, respectively. It also exhibited two more peaks at 934.9 and 954.8 eV for Cu(OH)_2_. PANI-Cu@BSA/rGO nanocomposites were successfully synthesized based on all of the above analyses.

### 3.2. Electrochemical Characterizations of Modified SPCEs

**Figure 4:**
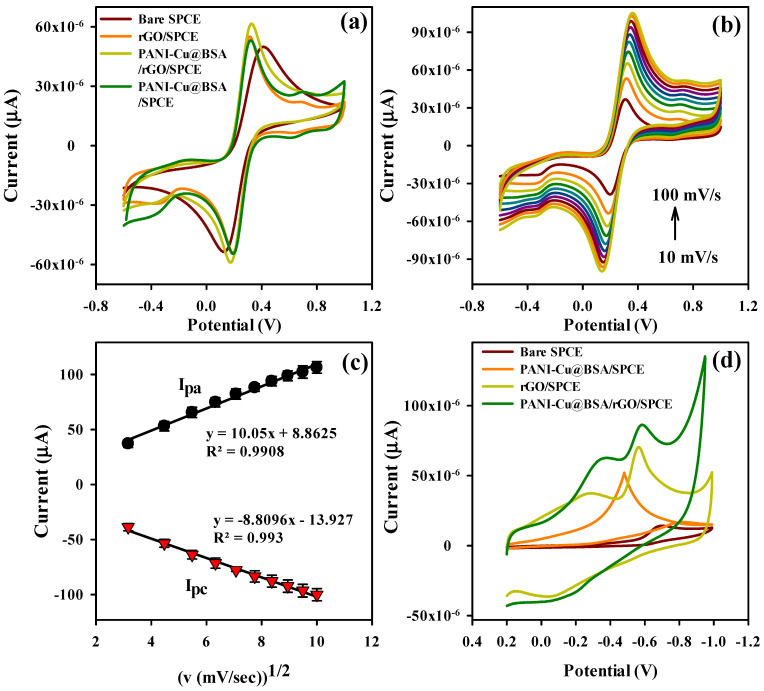
(**a**) CV profile of the bare SPCE, PANI-Cu@BSA/SPCE, rGO/SPCE, and PANI-Cu@BSA/rGO/SPCE; (**b**) CV profile of PANI-Cu@BSA/rGO/SPCE at various scan rates (10–100 mV/s) in 5 mM [Fe(CN)_6_]^3−/4−^/0.5 M KCl solution; (**c**) linear plot of current (I_pa_ and I_pc_) vs. (v (mV/s))^1/2^; (**d**) CV profile of the electrodes including bare, PANI-Cu@BSA, rGO, and PANI-Cu@BSA/rGO modified SPCEs in 150 µM DMZ containing 0.1 M PBS (pH 7) at 50 mV/s.

Cyclic voltammetry (CV) was used to estimate the electrochemical (EC) behavior of the bare SPCE, PANI-Cu@BSA/SPCE, rGO/SPCE, and PANI-Cu@BSA/rGO/SPCE in 5 mM [Fe(CN)_6_]^3−/4−^/0.5 M KCl solution at 50 mV/s (Figure 4a). The bare SPCE, PANI-Cu@BSA/SPCE, rGO/SPCE, and PANI-Cu@BSA/rGO/SPCE had redox peaks (anodic (I_pa_) and cathodic (I_pc_)) currents of I_pa_ = 49.9, 53.2, 55.1, and 61.5 µA and I_pc_ = 53.3, 54.0, 54.1, and 59.1 µA, respectively. Furthermore, the peak-to-peak separation (ΔEp) of the diverse modified electrodes resolved the electrocatalytic performances of the constructed sensor. The ΔEp of bare SPCE exhibited a much higher peak potential (0.29 V). Compared to bare SPCE, the ΔEp value for PANI-Cu@BSA/SPCE (0.14 V) and rGO/SPCE (0.15 V) showed a lower peak potential. Compared to all SPCEs, the PANI-Cu@BSA/rGO/SPCE composite displayed a lower peak potential (0.12 V) with enhanced redox behavior, signifying an enhanced interfacial redox process due to the existence of PANI-Cu@BSA on the rGO. On the other hand, the peak current ratios (I_pa_/I_pc_) of bare SPCE, PANI-Cu@BSA/SPCE, rGO/SPCE, and PANI-Cu@BSA/rGO/SPCE of 0.93, 0.98, 1.01, and 1.04 were acquired. Intriguingly, PANI-Cu@BSA/rGO/SPCE showed good reversible behavior with higher redox-peak currents and smaller peak separations. Additionally, the PANI-Cu@BSA/rGO/SPCE composite showed a synergistically enhanced peak current response compared with other modified electrodes due to the high electrical conductivity and superior electrochemical activity of rGO in the composite materials. Moreover, CV was used to estimate the electrode kinetics with different scan rates (10–100 mV/s) with the existence of diffusible redox pairs in the ([Fe(CN)_6_]^3−/4−^/0.5 M KCl) solution (Figure 4b). As shown in Figure 4b, I_pa_ and I_pc_ were linearly increased with the increase in the scan rate. Figure 4c shows the plots of the square root of the scan rate ((v (mV/s))^1/2^) vs. I_pa_ and I_pc_, and the regression Equations (1) and (2) are as follows:

I_pa_ (µA) = 10.058096 (v (mV/s))^1/2^ + 8.862 (1)

I_pc_ (µA) = −8.8096 (v (mV/s))^1/2^ − 13.927 (2)

These results suggest that a diffusion-controlled process took place on the modified electrode surface. Moreover, the electroactive surface area (A) was calculated based on the Randles–Sevcik Equation (3):

I_pa_ = (2.691 × 10^5^) n^3/2^D^1/2^v^1/2^ AC (3)

where I_pa_ and v are the oxidation peak current (µA) and scan rate (mVs^−1^), respectively; and C, n, and D represent the concentration (mol cm^−3^), number of electrons participating, and diffusion coefficient (7.2 × 10^−6^ cm^2^S^−1^), respectively. Based on the slope value obtained from the plot of I_pa_ vs. (v (mV/s))^1/2^, the ‘A’ of the different electrodes was calculated. Using Equation (3), the bare SPCE, PANI-Cu@BSA/SPCE, rGO/SPCE, and PANI-Cu@BSA/rGO/SPCE were calculated to be 0.118 cm^2^, 0.134 cm^2^, 0.158 cm^2^, and 0.196 cm^2^, respectively. 

### 3.3. Electrocatalytic Reduction of DMZ on PANI-Cu@BSA/rGO/SPCE

The CV technique was used to study the electrocatalytic reduction of DMZ using different modified SPCEs. Figure 4d shows the CV curves of the bare SPCE, PANI-Cu@BSA/SPCE, rGO/SPCE, and PANI-Cu@BSA/rGO/SPCE in 150 µM of DMZ (pH = 7 (0.1 M)) at 50 mV/s. The study found that bare SPCE had a low level of current intensity in the reduction of DMZ at a high negative potential (−0.68 V (vs. Ag/AgCl)) due to the sluggish electron transfer rate, whereas the DMZ reduction peak was found in the CV current response of PANI-Cu@BSA/SPCE (−52.4 µA) at a negative potential of −0.48 V (vs. Ag/AgCl). 

**Scheme 2:**
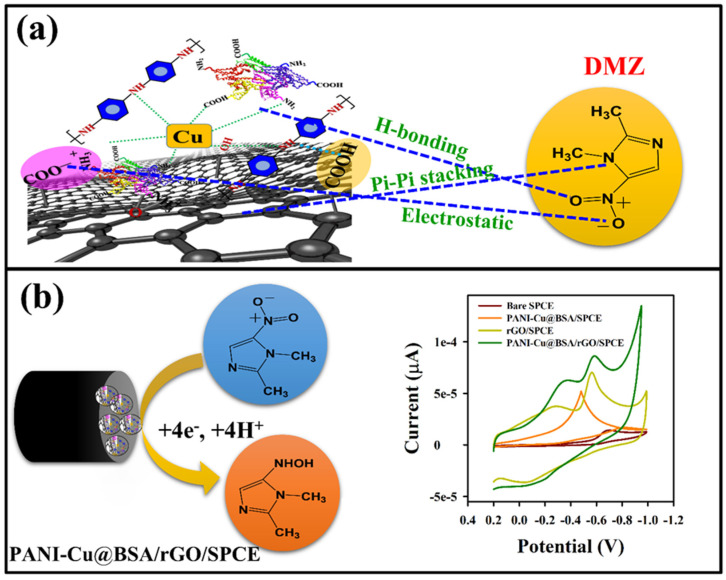
(**a**) Schematic illustration of possible interaction between the DMZ and PANI-Cu@BSA/rGO and (**b**) the plausible electroreduction mechanism of DMZ on the PANI-Cu@BSA/rGO/SPCE.

Compared with bare SPCE and PANI-Cu@BSA/SPCE, the rGO/SPCE result revealed that DMZ had a higher reduction current (−70.1 µA) at a lower negative potential of −0.56 V (vs. Ag/AgCl). Moreover, PANI-Cu@BSA/rGO/SPCE showed an increased I_pc_ current intensity (−86.9 µA) at a lower reduction potential of −0.57 V. These values were 5.9, 1.6, and 1.2 times higher compared with bare SPCE, PANI-Cu@BSA/SPCE, and rGO/SPCE, respectively, which demonstrates that the newly synthesized PANI-Cu@BSA/rGO/SPCE had good EC activity and a synergistic effect on the DMZ detection. The synergistic catalyst activity of PANI-Cu@BSA/rGO/SPCE was enhanced by the conductive rGO and PANI-Cu@BSA present in the composite, resulting in improved electrocatalytic activity of PANI-Cu@BSA/rGO/SPCE. Thus, the composite electrocatalyst allowed for highly sensitive and selective DMZ sensing. A plausible electrocatalytic reduction mechanism for DMZ on PANI-Cu@BSA/rGO/SPCE is shown in Scheme 2b. The EC reduction peak intensity appeared due to the conversion of the –NO_2_ groups in DMZ to –NHOH groups via 4e^−^ and 4H^+^ EC processes [43]. Due to the synergetic electrocatalytic properties, there could be some interactions between PANI-Cu@BSA/rGO and DMZ. DMZ’s benzene ring had pi–pi stacking interactions with the benzene structure of rGO and PANI. Through hydrogen bonds, DMZ interacted with –COOH groups in BSA. Additionally, –NO_2_ groups in DMZ interacted with –NH_3_ groups in PANI-Cu@BSA/rGO via electrostatic interactions. Scheme 2a illustrates the possible interaction mechanism between DMZ and PANI-Cu@BSA/rGO. 

**Figure 5:**
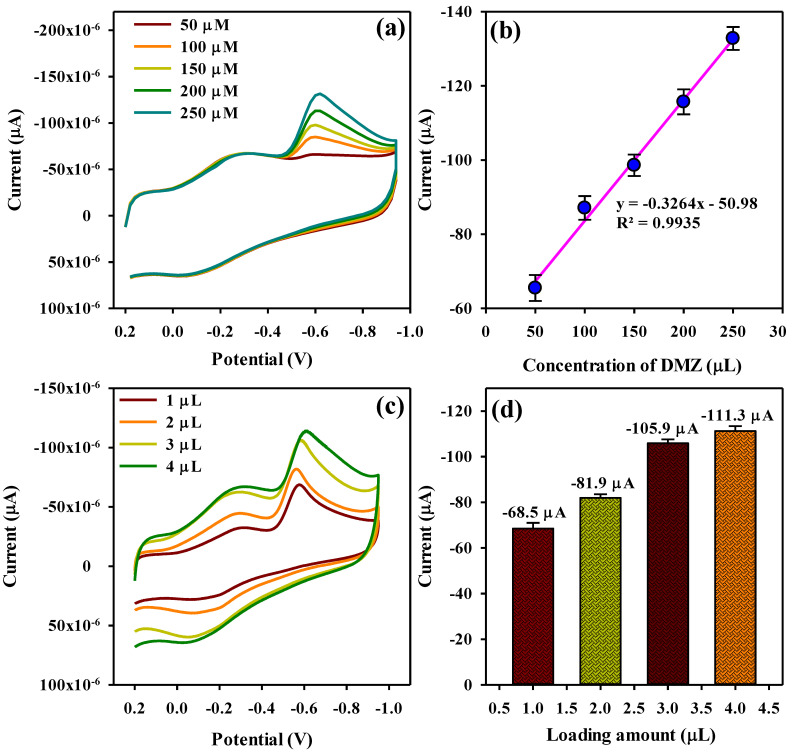
(**a**) CV profile of PANI-Cu@BSA/rGO/SPCE in different concentrations of DMZ (50–250 µM) at 50 mV/s, (**b**) linear plot of current vs. concentration of DMZ, (**c**,**d**) CV profile of the effect of loading level of PANI-Cu@BSA/rGO on the SPCE surface for the detection of DMZ at 50 mV/s, and (**d**) calibration plot of current vs. loading amount (µL).

The antifouling property of a prepared electrode is important for the detection of a target analyte, and it can be studied by varying the concentration of DMZ used in the CV measurements. Figure 5a depicts the EC behavior of PANI-Cu@BSA/rGO/SPCE with DMZ at different concentrations (50–250 µM) established in 0.1 M PBS at a scan rate of 50 mV/s. As presented in Figure 5a, increasing the concentration of DMZ increased the reduction peak current. Figure 5b shows a linear plot of peak current for the reduction of DMZ at different concentrations, and the linearity regression gave R^2^ = 0.9935, I_pc_ (µA) = −0.3264, and Con_µM_ = −50.98. Based on these results, PANI-Cu@BSA/rGO/SPCE demonstrated high electrocatalytic activity for DMZ detection. 

CV responses of the effect of the loading amount of PANI-Cu@BSA/rGO ranging from 1 to 4 µL at pH 7 (0.1 M PBS) on DMZ detection were also revealed (Figure 5c). When increasing the loading of electrode material on the SPCE from 1 to 4 µL, the I_pc_ current intensity of DMZ also increased. Loading of 4 µL resulted in the maximum current (Figure 5d), hence 4 µL of electrode material was found to be the optimal loading amount in DMZ electroreduction. 

**Figure 6:**
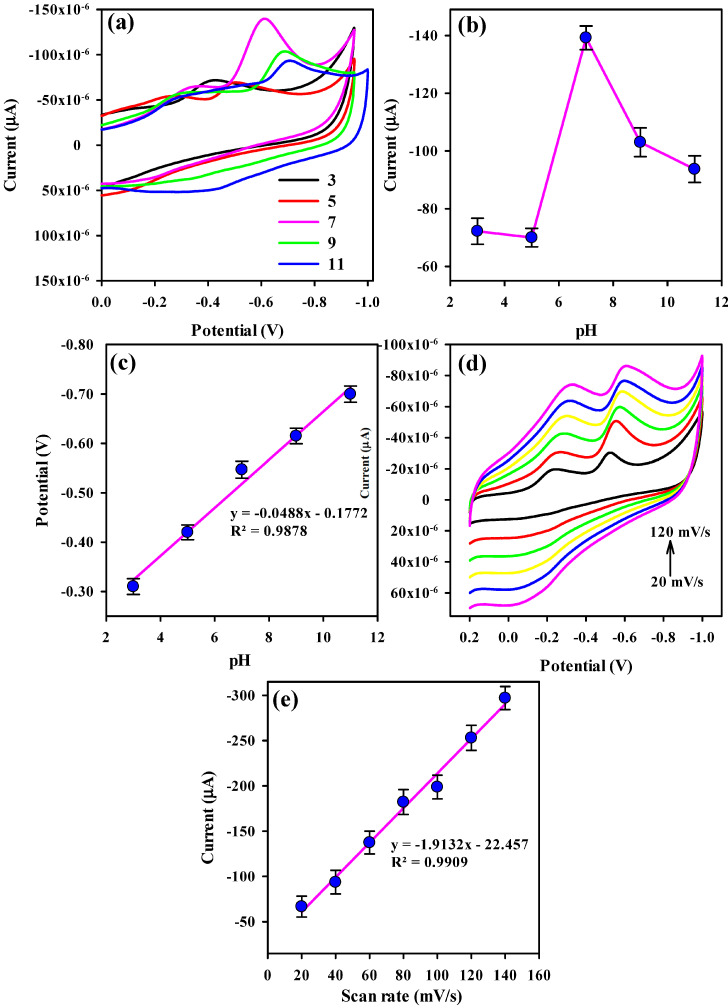
(**a**) CV profile of PANI-Cu@BSA/rGO/SPCE in various pH ranges from 3 to 11 at 50 mV/s, (**b**) linear plot of current vs. pH, (**c**) linear plot of potential vs. pH values, (**d**) CV profile of PANI-Cu@BSA/rGO/SPCE at various scan rates (0.1 M PBS) from 20 to 120 mV/s, and (**e**) linear relationship between current and scan rate.

The influence of pH on the reduction of DMZ on the PANI-Cu@BSA/rGO/SPCE was investigated in the presence of 150 μM of DMZ with different pH levels from 3 to 11 by using CV at 50 mV/s (Figure 6a). The reduction peaks’ current increased with an increase in pH from 3 to 7 and then decreased when increasing the pH from 7 to 11 (Figure 6b). As the pH increased, the reduction peak potential moved to the more negative side, demonstrating involvement of protons in the EC process of DMZ. The maximum peak current was observed at pH 7 because at this level, the electrolyte facilitated electrons moving at their highest speed toward the electrode surface [43]. Consequently, the PBS solution with pH 7 was preferred to be the supporting electrolyte for the following reasons: (i) it had a clearly defined reduction peak current with enriched EC behavior, and (ii) it simulated a physiological environment [43]. EC measurements were conducted at pH 7 for the detection of DMZ based on these results. Furthermore, analyses were conducted on the cathodic peak potential as a function of pH (Figure 6c), and the linear regression equation was found to be Epa (V) = −0.0488 pH − 0.1772 (R^2^ = 0.9878). The slope was found to be 48 mV/pH, indicating that the process of reducing DMZ on the PANI-Cu@BSA/rGO/SPCE involved an equal number of electron and proton exchange reactions [39,44].

For the purpose of confirming the kinetic mechanism of PANI-Cu@BSA/rGO/SPCE, the effect of the scan rate on the DMZ peak reduction current was measured at different scan rates in 0.1 M PBS (pH 7), as shown in Figure 6d. When increasing the scan rate from 20 to 120 mV, the I_p_ also increased linearly with the linear regression equation of Ipc = −1.9132 mV/s − 22.457 (R^2^ = 0.9909) (Figure 6e). This data demonstrates that the EC detection of DMZ on PANI-Cu@BSA/rGO/SPCE was controlled by an adsorptive process onto the sensor surface. Furthermore, the linear plot of the peak potential and log of the scan rate is shown in Figure S3. The electron transfer number was determined by utilizing the Laviron Equation (4):

E_p_ = E^0^ + (RT/αnF) ln(RTk^0^)/αnF) + (RT/αnF)lnv (4)

where v is the scan rate, F is the Faraday constant, n is the number of electrons transferred, k^0^ is the heterogeneous rate constant, α is the electron transfer coefficient, T is the temperature (K), E_p_ represents the reduction peak potential, and E^0^ is the formal potential. The n (3.81~4) was estimated from the slope in Figure S3. From the result, we affirmed that the electroreduction of DMZ on PANI-Cu@BSA/rGO/SPCE involved a 4e^−^ and 4H^+^ process.

### 3.4. Quantitative Determination and Selectivity Studies of DMZ

**Figure 7:**
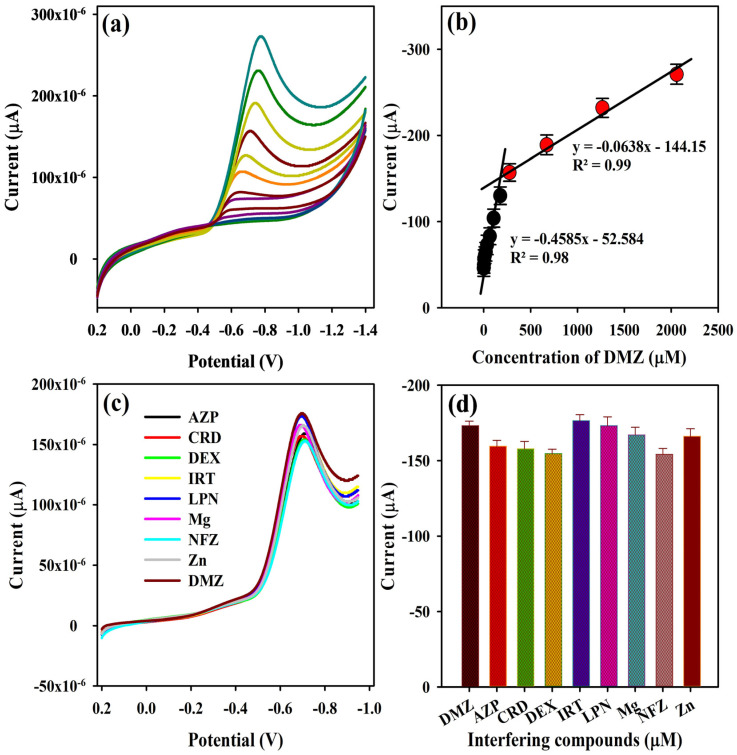
(**a**) LSV profile of PANI-Cu@BSA/rGO/SPCE at different concentrations of DMZ from 0.79 to 2057 µM at 50 mV/s, (**b**) calibration plot of DMZ vs. cathodic peak current (μA) (*n* = 3), (**c**) LSV profile of PANI-Cu@BSA/rGO/SPCE on the inferring effect of DMZ with various co-interfering compounds (AZP, CRD, DEX, IRT, LPN, Mg^2+^, Zn^2+^, and NFZ) at 50 mV/s, and (**d**) corresponding plot of current vs. interfering compounds.

By using the linear sweep voltammetry (LSV) technique, the quantitative determination of DMZ’s electrochemical response on PANI-Cu@BSA/rGO/SPCE was investigated (Figure 7a). Due to the synergistic activity and non-covalent interaction between PANI-Cu@BSA and rGO, increasing the DMZ concentration resulted in an increasing I_pc_ intensity, which illustrates the electroanalytical performance for the reduction of DMZ. A linear dependence was observed between the I_pc_ and DMZ concentration in both low and high ranges of concentration. The current is plotted against the concentration in Figure 7b. 

A rapid response was observed when lower concentrations of DMZ were added to the electrode surface. In contrast, higher concentrations of DMZ caused much slower molecule movement. These irregular responses led to the formation of two linear regions [43]. Using the plot (Figure 7b), the range for lower linear regression (0.79–175 μM) was estimated to be I_pc_ (µA) = −0.4585 ConDMZ (µM) − 52.584, with a correlation coefficient (R^2^) of 0.98. The higher linear regression (275–2057 μM) was estimated to be I_pc_ (µA) = −0.0638 ConDMZ (µM) − 144.15, with a correlation coefficient (R^2^) of 0.99. The reduction of DMZ occurred through the direct transfer of electrons between the electrode surfaces and DMZ molecules. Table 1 shows the comparison of the analytical performance of the PANI-Cu@BSA/rGO/SPCE with recently reported electroanalytical performances based on DMZ EC detection.

The LOD of the PANI-Cu@BSA/rGO/SPCE toward DMZ was estimated using Equation (5) below:

LOD = nσ/s(5)

where “n” represents a triplicate, “σ” is the standard deviation of three blank measurements, and “s” is the slope value of the linear plot.

A lower LOD (1.78 nM) and a wide dynamic linear range (0.79 to 2057 μM) were achieved with the prepared sensor when compared to other reports.

Selectivity had a crucial role in evaluating the feasibility of using PANI-Cu@BSA/rGO/SPCE for the detection of DMZ with coexisting interfering compounds. The following compounds were used as possible coexisting compounds with DMZ: azathioprine (AZP), carbendazim (CRD), dexamethasone (DEX), irinotecan (IRT), lopinavir (LPN), magnesium (Mg^2+^), zinc (Zn^2+^), and nitrofurazone (NFZ). The LSV response of interfering effect on the PANI-Cu@BSA/rGO/SPCE toward DMZ detection in 0.1 M PBS (50 μM) was investigated along with the other interfering compounds (AZP, CRD, DEX, IRT, LPN, Mg^2+^, Zn^2+^, and NFZ) (Figure 7c). The impact of interfering compounds on the I_pc_ peak current was minimal, with less than 5% deviation, which demonstrated that PANI-Cu@BSA/rGO/SPCE has an excellent interfering effect for DMZ detection (Figure 7d). During reduction, negative charge transfer may be accelerated due to the absorption of the target DMZ species on the PANI-Cu@BSA/rGO/SPCE surface. Additionally, the presence of amide, carboxylic, and amino groups in PANI-Cu@BSA/rGO/SPCE allowed for the formation of various types of chemical interactions with the target DMZ molecules, such as hydrogen bonding, electrostatic interactions, and pi–pi interactions. These interactions facilitate easy transfer of electrons. As a result, we concluded that the sensor prepared for DMZ detection was not affected by potentially coexisting compounds. 

### 3.5. Repeatability and Stability Studies

The repeatability of seven measurements on the PANI-Cu@BSA/rGO/SPCE was estimated in 20 μM of DMZ by LSV (Figure S4a). As shown in Figure S4b, a bar graph shows changes in I_pc_ for seven measurements. Based on the RSD calculation (1.47%), the PANI-Cu@BSA/rGO/SPCE sensor demonstrated good repeatability. To assess the stability of the sensor, the I_pc_ current response of DMZ detection on the PANI-Cu@BSA/rGO/SPCE was measured over 20 cycles. PANI-Cu@BSA/rGO/SPCE demonstrates superior stability, as illustrated in Figure S4c,d. This indicates that the I_pc_ current responses are not greatly affected (93.8%) at the 20th run. The storage stability of the PANI-Cu@BSA/rGO/SPCE was revealed by LSV response toward 50 μM DMZ as presented in Figure S5. The stability of PANI-Cu@BSA/rGO/SPCE was demonstrated by storing the electrode at room temperature and recording its LSV scans at 5-day intervals. The DMZ’s current response maintained 93% of its initial value following a 30-day period, illustrating the stability achieved in detecting DMZ on the PANI-Cu@BSA/rGO/SPCE.

### 3.6. Real Sample Analysis

The applicability and viability of our developed PANI-Cu@BSA/rGO were inspected by detecting DMZ in human and rat blood serum samples and egg samples. The real samples were carried out by using the standard addition method. To prepare the samples for analysis, human and rat blood serums were diluted (10 times) in 0.1 M PBS (pH 7). For real sample analysis of egg, 2.0 g was transferred to a centrifuge tube and sonicated for proper dispersion. The real samples were obtained by using centrifugation with 10% trichloroacetic acid (5 mL) at 3000 rpm. The dilution process of the supernatant liquid took place with ethanol (10 mL). The human, rat blood serum, and egg samples were DMZ-free; thus, the known concentration of DMZ (0.01, 0.1, 1, 5, 10, or 15 μM) was added into the diluted real samples. The recovery percentages (ranging from 90–99.6%) for the human, rat blood serum, and egg samples are shown in Table 2. Moreover, the RSD value of PANI-Cu@BSA/rGO/SPCE was found to be 1–4%, indicating that the method was more effective for detecting DMZ in human blood, rat blood serum, and egg samples. Hence, our constructed PANI-Cu@BSA/rGO/SPCE is reliable for real-time detection.

## 4. Conclusions

In conclusion, a PANI-Cu@BSA/rGO nanocomposite was prepared by using one-pot biomimetic mineralization followed by an ultrasonication method for DMZ detection. The prepared nanocomposite was analyzed using different techniques that included FESEM, TEM, FTIR, DLS, Raman, and XPS. Selective and sensitive DMZ EC detection was performed using CV and LSV on the hybrid PANI-Cu@BSA/rGO nanocomposite modified on an SPCE surface. PANI-Cu@BSA/rGO/SPCE possessed a low detection limit, a high sensitivity, and a linear range of 1.78 nM, 5.96 μA μM^−1^ cm^−2^, and 0.79 to 2057 μM, respectively. It also showed excellent stability and selectivity as well as good practicability for the detection of DMZ in biological fluid and egg samples. The technique of using BSA cross-linked conducting polymers to create antifouling electrochemical sensing interfaces can be applied to the creation of various biosensors for detecting various targets in biological samples. By using the fabricated EC sensor, routine grade control of large-scale drugs and biological fluid analysis can be carried out without the need for expensive instruments or intricate sample preparation. 

## Figures and Tables

**Table 1:** Comparison of this method with other reported methods for the detection of DMZ.

Modified Electrode Material	Method	Linear Range (μM)	LOD	Ref.
Se-Co_3_O_4_@GO-NC/GCE	DPV	0.02–83.72	3.4 nM	[5]
DM/Nd_2_Sn_2_O_7_/GCE	DPV	0.01-1453	6.0 nM	[11]
Mn-SnO@rGO/GCE	DPV	0.009–1291	2.0 nM	[43]
Cu_2_O/ErGO/GCE	DPV	0.03–0.15	3.6 nM	[45]
PARG-GQDs/GCE	DPV	0.03–0.15	3.6 nM	[46]
PANI-Cu@BSA/rGO/SPCE	LSV	0.79–2057	1.78 nM	This work

**Table 2:** Real-time detection of DMZ in human blood serum, rat blood serum, and egg samples for PANI-Cu@BSA/rGO/SPCE.

Samples	Loaded (μM)	Found (μM)	Recovery (%)	RSD (%)
Human blood serum	0.01	0.009	90.0	2.16
	0.1	0.095	95.0	2.38
	1	0.97	97.0	1.32
	5	4.6	92.0	2.12
	10	10.0	100.0	1.58
	15	14.5	96.6	3.21
Rat blood serum	0.01	0.0101	101.0	1.91
	0.1	0.099	99.0	1.81
	1	1.01	101.0	2.16
	5	5.0	100.0	1.51
	10	9.8	98.0	2.98
	15	15.1	100.6	3.12
Egg	0.01	0.0099	99.0	2.08
	0.1	0.098	98.5	1.52
	1	0.97	97.0	2.48
	5	4.9	98.0	1.85
	10	10	100	3.48
	15	14.95	99.6	2.56

## Data Availability

The data presented in this study are available on request from the corresponding author.

## Supplementary Materials

The following supporting information can be downloaded at: https://www.mdpi.com/article/10.3390/polym16010162/s1, Figure S1: TEM image of rGO; Figure S2: (a–e) Elemental mapping and (f) EDX image of PANI-Cu@BSA/rGO; Figure S3: Plot of potential vs. log of scan rate; Figure S4: (a,b) LSV profile of repeatability of PANI-Cu@BSA/rGO/SPCE towards the detection of DMZ at 7 consecutive measurements and the corresponding plot of current vs. the number of measurements, (c,d) LSV profile of operational stability of PANI-Cu@BSA/rGO/SPCE on the detection of DMZ at 50 mV/s and plot of current vs. Number of runs; Figure S5: Storage stability of the sensor.
