# Supplementary material for: Protein-Aided Synthesis of Copper-Integrated Polyaniline Nanocomposite Encapsulated with Reduced Graphene Oxide for Highly Sensitive Electrochemical Detection of Dimetridazole in Real Samples
Source: Polymers (Basel). 2024 Jan 4;16(1):162. doi: 10.3390/polym16010162 (PMC10781186; doi:10.3390/polym16010162)
Supplement: Supplementary file 1 [file polymers-16-00162-s001.zip › polymers-2753141-supplementary.pdf]

Supporting file

Protein-Aided Synthesis of Copper-Integrated Polyaniline Nanocomposite Encapsulated with Reduced Graphene Oxide for Highly Sensitive Electrochemical Detection of Dimetridazole in Real Samples

Kartik Behera [1], Bhuvanenthiran Mutharani [1], Yen-Hsiang Chang [2], Monika Kumari [3] and Fang-Chyou Chiu [1,2,*]

[1] Department of Chemical and Materials Engineering, Chang Gung University, Taoyuan 333, Taiwan; b.kartik1991@gmail.com (K.B.); mutharani@gmail.com (B.M.)
[2] Department of General Dentistry, Chang Gung Memorial Hospital, Taoyuan 333, Taiwan; chy4d25@cgmh.org.tw
[3] Institute of Cellular and Organismic Biology, Academia Sinica, Taipei 115, Taiwan; mkumari.biotech@gmail.com
* Correspondence: maxson@mail.cgu.edu.tw

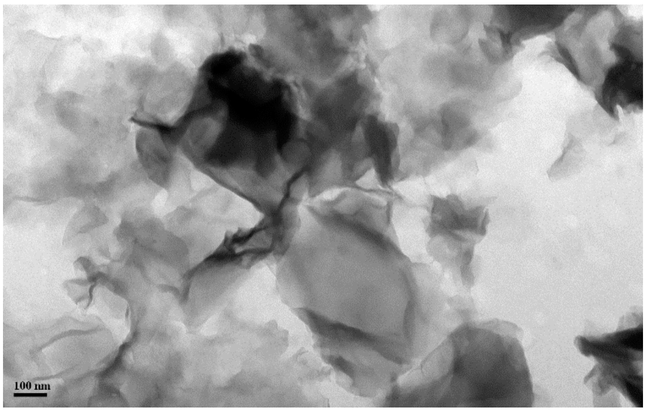

Figure S1. TEM image of rGO.

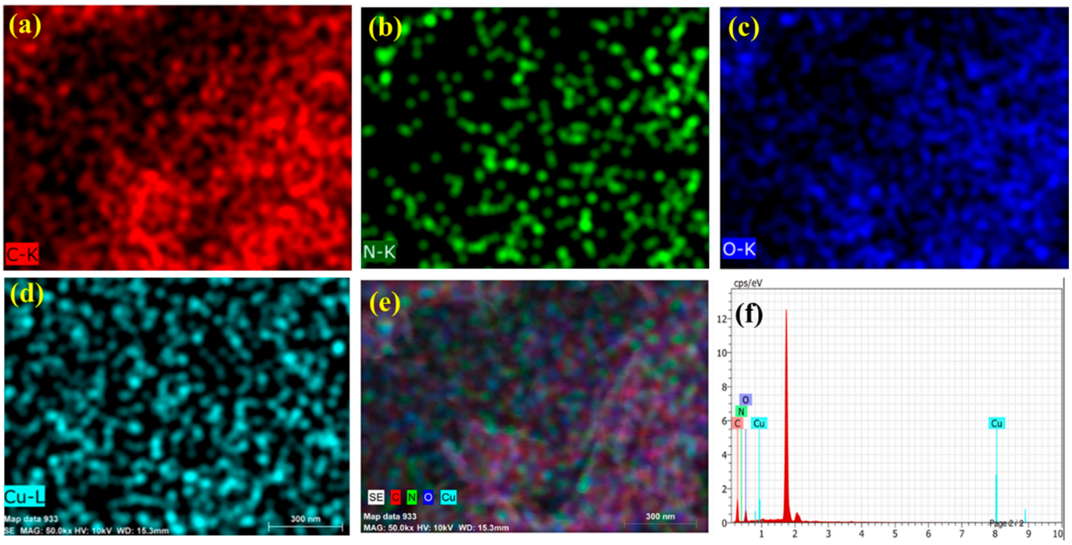

Figure S2. (a-e) Elemental mapping and (f) EDX image of PANI-Cu@BSA/rGO.

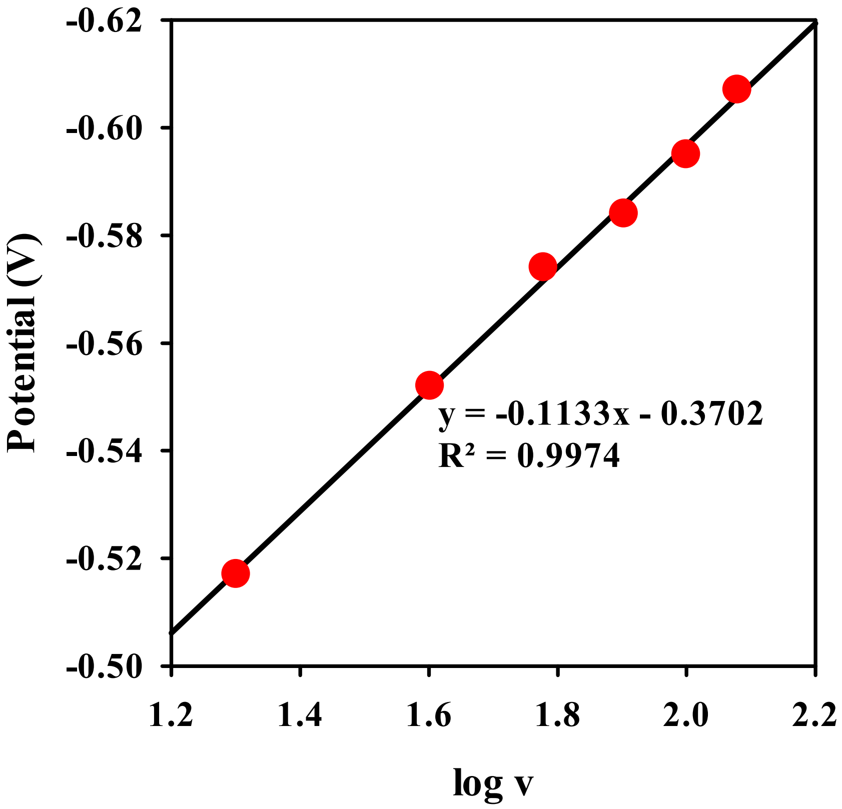

Figure S3. Plot of potential vs. log of scan rate.

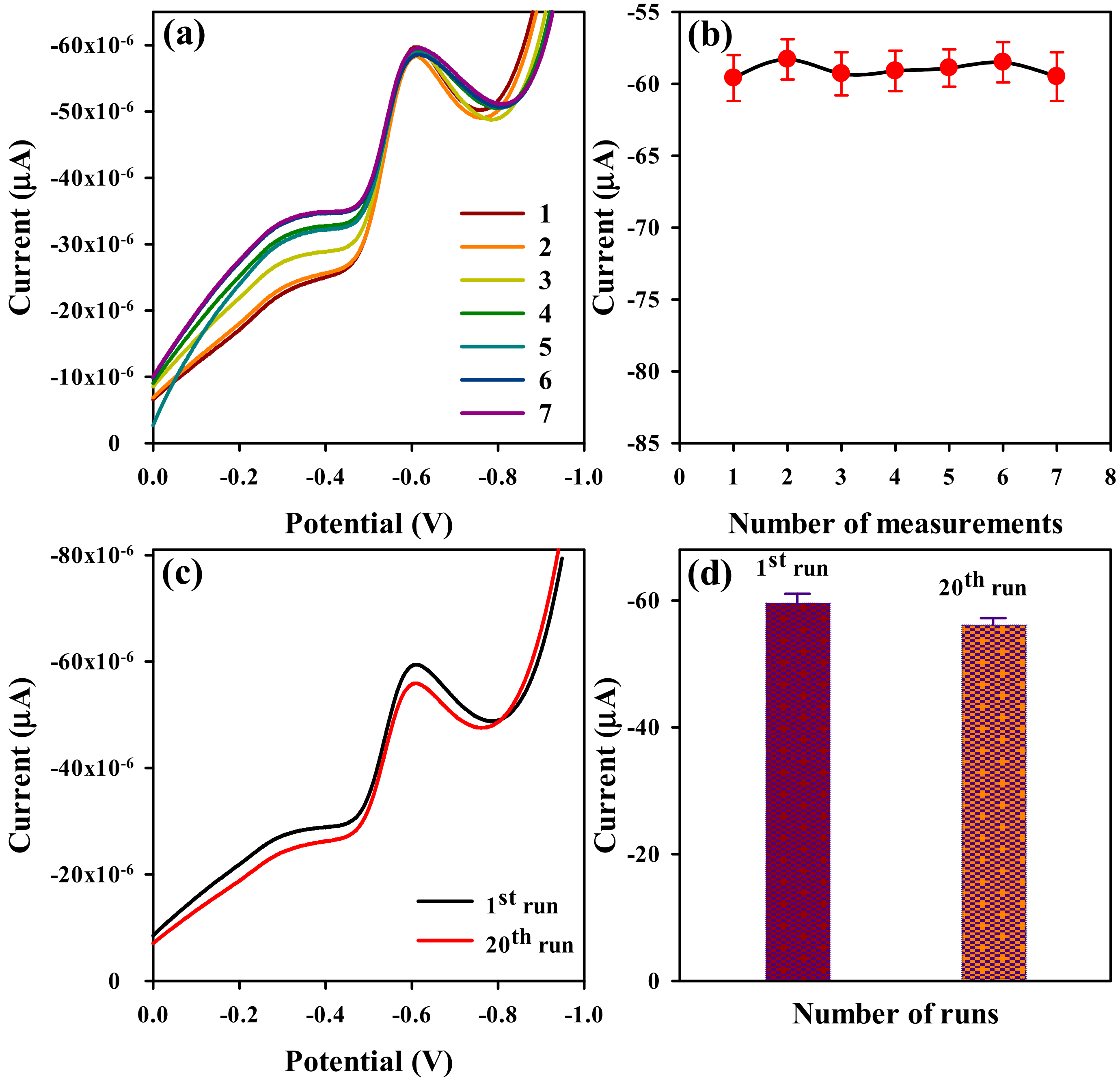

Figure S4. (a and b) LSV profile of repeatability of PANI-Cu@BSA/rGO/SPCE towards the detection of DMZ at 7 consecutive measurements and the corresponding plot of current vs. the number of measurements, (c and d) LSV profile of operational stability of PANI-Cu@BSA/rGO/SPCE on the detection of DMZ at 50 mV/s and plot of current vs. Number of runs.

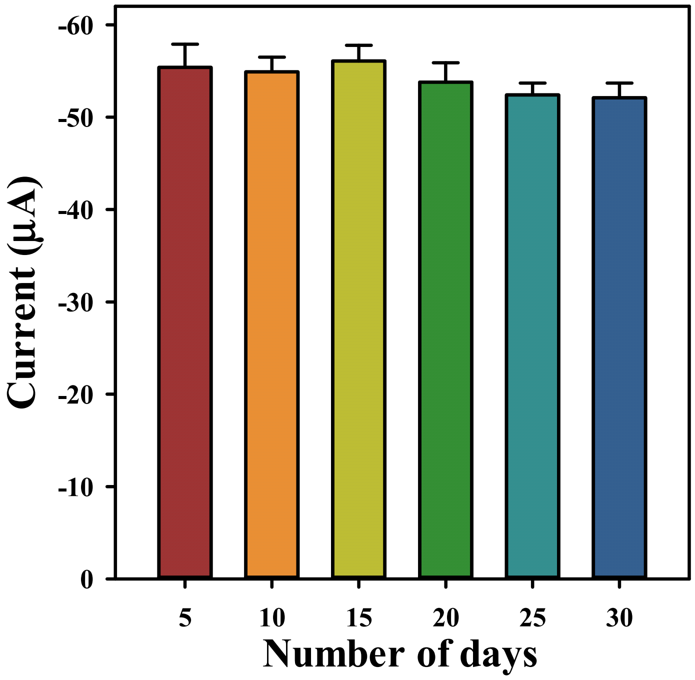

Figure S5. Storage stability of the sensor.
